# CircRNA-miRNA-mRNA regulatory network in high-altitude hypobaric hypoxia-induced hearing impairment and hearing acclimatization

**DOI:** 10.1016/j.bjorl.2024.101557

**Published:** 2025-01-27

**Authors:** Danzeng Awang, Kanzi Danzeng, Tianheng Wang, Quzong Deji, Mengting Huang, Hailong Ren, Xinzhu Liu, Binghan Zhao, Lanzi Gongga

**Affiliations:** a Medical College, Tibet University, Department of Clinical Medicine, Lhasa, China; b Lhasa People's Hospital, Children's Surgery Department, Lhasa, China; c Health Service Center of Jiri Street Office, Chengguan District, Lhasa, China; d Tibet University, Medical College, Lhasa, China

**Keywords:** circRNAs, High altitude, Hypobaric hypoxia, Hearing acclimatization, Hearing impairment

## Abstract

**Objective:**

High altitude hypobaric hypoxia can induce hearing impairment and hearing acclimatization, but few studies have been performed to decipher the potential transition between the two states. To decipher transition-related circular RNAs (circRNAs)-microRNAs (miRNAs)-messenger RNA (mRNAs) regulatory network.

**Methods:**

Wistar rats were airlifted from plain to high altitude and maintained for 30 days and 60 days. Hearing acclimatization was determined using the Auditory Brainstem Response (ABR) test. Cochlea tissues were isolated, and high-throughput circRNA analysis and mRNAs-sequencing were performed. Differentially Expressed circRNAs (DEcircRNAs) and Differentially Expressed mRNAs (DEmRNAs) were obtained, and circRNA-miRNA and miRNA-mRNA regulation were predicted. A circRNA-miRNA-mRNA competing endogenous RNA (ceRNA) network was also constructed. The DEmRNAs in this network were functionally annotated using Gene Ontology and Kyoto Encyclopedia of Genes and Genomes analyses based on Metascape.

**Results:**

The ABR assay indicated that hearing impairment happened on day 30 and hearing acclimatization occurred on day 60. Hearing impairment-related circRNAs (64 upregulated and 147 downregulated) and genes (572 upregulated and 757 downregulated) were identified. Hearing acclimatization-related circRNAs (79 upregulated and 142 downregulated) and genes (690 upregulated and 751 downregulated) were also identified. Hearing impairment and hearing acclimatization ceRNA networks were also constructed after integrating the predicted miRNA regulation analyses. Anterograde trans-synaptic signaling (GO:0098916) and negative regulation of cellular response to growth factor stimulus (GO:0090288) were regulated by hearing impairment ceRNA networks, and embryonic organ development (GO:0048568) was regulated by hearing acclimatization ceRNA networks.

**Conclusion:**

Hearing impairment- and hearing acclimatization-associated circRNAs and ceRNA networks were identified, which contribute new knowledge to our understanding of acclimatization transition.

## Highlights

• Hearing impairment- and acclimatization-related circRNAs and genes were identified.
• Hearing impairment and hearing acclimatization ceRNA networks were constructed.
• Hearing impairment ceRNA networks regulated anterograde trans-synaptic signaling.
• Hearing impairment ceRNAs regulated the negative response to growth factor stimuli.
• Hearing acclimatization ceRNA networks regulated embryonic organ development.

## Introduction

At high altitudes, a reduction in barometric pressure and consequent drop in partial oxygen pressure is defined as hypobaric hypoxia,^1^, ^2^ which can cause alterations in cerebral, cardiovascular, respiratory, auditory, and hormonal systems.^3^, ^4^ Persons exposed to hypobaric hypoxia will suffer acute low-frequency and high-frequency hearing loss when they arrive at the high altitude, and then resume normal hearing four days later.^5^ In mice, high-altitude hypoxia acclimatization can improve developmental and cognitive deficits induced by acute fetal hypoxia.^6^

Although humans can acclimate to intermittent or chronic hypobaric hypoxia, altitude sickness, especially high-altitude cerebral edema and high-altitude pulmonary edema, remains potentially lethal.^7^, ^8^ Acetazolamide, dexamethasone, and montelukast are recommended for the treatment of altitude sickness, but the clinical utilization of these drugs is limited by their various adverse effects, such as headache, cardiopalmus, osteoporosis, sensory abnormalities, and increased risk of infection. Thus, there is an urgent need to develop alternative therapies to prevent or acclimate to these conditions.

Circular RNAs (circRNAs) are transcribed from the intron or exon region of a gene as noncoding RNA with closed circular structures, which make circRNAs resistant to RNA exonucleases.^9^, ^10^, ^11^ Although the generation and function of circRNAs are not fully clear, a recent investigation demonstrated that circRNAs function in the initiation and progression of human disorders.^12^, ^13^ Functionally, circRNAs can sponge microRNA (miRNA) to indirectly regulate the post-transcriptional expression of miRNA-targeted mRNAs, which are defined as competing endogenous RNAs (ceRNAs).^14^, ^15^ However, the potential role of the circRNA/miRNA/mRNA ceRNA network in high-altitude hypobaric hypoxia-induced hearing impairment and hearing acclimatization has not been fully explored.

**Fig. 1:**
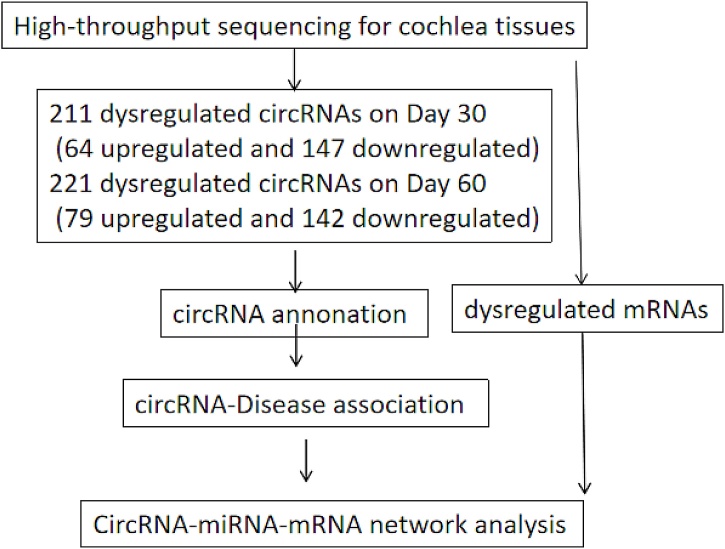
Workflow of stage-related circRNA identification and competing endogenous RNA (ceRNA) network analysis.

Whether prolonged exposure to high-altitude hypobaric hypoxia can induce hearing acclimatization is an interesting question without a distinct conclusion. In this study, Wistar rats were airlifted from a plain to a high altitude position and maintained in a relatively controlled environment. Under these experimental conditions, hearing impairment and hearing acclimatization processes develop, allowing the investigation of stage-related circRNA and circRNA/miRNA/mRNA ceRNA network analyses (Fig. 1) to determine the potential mechanisms underlying these processes.

## Methods

### High-altitude hypobaric hypoxia rat model

Wistar rats (8-week-old) ordered from Chengdu DaShuo Experimental Animals Co., LTD (China) were airlifted to Jilin University (about 200 m asl, n = 5; Ctrl group) or Tibet University (Lhasa, Tibet Autonomous Region, China; about 3,700 m asl, for 30-days or 60-days, n = 5; D30 group and D60 group, respectively). The entire experimental protocol was approved by the Medical Ethics Committee of Hospital (No. 2018-XZDX-006) and conducted following the ARRIVE guidelines. All methods were performed in accordance with the relevant guidelines and regulations.

### Auditory brainstem response (ABR) testing

After anaesthetization with 0.005 mg/g xylazine and 0.1 mg/g ketamine, rats were put in the sound-attenuating chamber (Acoustics). Subdermal needle electrodes were inserted behind the non-stimulated and tone-burst-stimulated ears and at the vertex position to detect the cochlea-evoked response (8, 16, and 32 kHz). The recorded responses were amplified by the SA1 stereo power amp (Tucker-Davis Technologies) and filtered through BioSig software.

### circRNA prediction and differential expression analysis

Total RNAs in the cochlea tissue extracted using RNeasy Mini Kit (Qiagen, Hilden, Germany) were treated with RNase R (Epicenter) and Epicenter Ribo-Zero rRNA Removal Kit (Illumina) to remove linear and ribosomal RNAs. Libraries were constructed using the TruSeq® Stranded Total RNA HT/LT Sample Prep Kit (Illumina) by Shanghai Ouyi Biomedical Co., Ltd. After 3’ adaptor and N3-containing sequence trimming, low-quality paired-end reads or short reads (less than 50 bp) were removed using Cutadapt v2.3 software.^16^ Then, high-quality reads were aligned to mRatBN v7.2 using Bowtie2 software.^17^ Paired chiastic clipping signal scanning was performed using CIRI.^18^ Based on junction reads and GT-AG cleavage signals, circRNA sequences were predicted. DESeq V3.15 software was utilized to standardize the number of circRNA counts for each sample and to estimate expression levels with baseMean values. The negative binomial distribution test was adopted to perform a significance test on the number of reads (fold-change >2.0, *p*-value < 0.05) to acquire differentially expressed circRNAs (DEcircRNAs).

### mRNA expression and differential expression analysis

Total RNA was isolated from rat cochlea tissues, followed by data preprocessing and genomic alignment by Shanghai Ouyi Biomedical Co., Ltd. Libraries were generated using TruSeq Stranded Total RNA with Ribo-Zero Gold, which were further sequenced on the Illumina HiSeq™ 2500 platform to generate Fastq format raw data. SortMeRNA software^19^ was used to remove the residual rRNA sequence, and Trimmatic software^17^ was utilized to filter the adapter, fuzzy base, and low-quality reads to obtain clean reads. Read mapping and transcript expression level quantification were performed using the HISAT2, StringTie, and DESeq2 workflows.^18^ to align with the mRatBN7.2 genome, quantify transcripts, and perform Differentially Expressed Gene (DEG) analysis using the criteria of |log2FC| > 1.0 and *p* < 0.05.

### miRNA prediction and circRNA-miRNA-mRNA network construction

Arraystar microRNA prediction software^20^ was utilized to predict miRNAs downstream of DEcircRNAs. TargetScan^21^ and miRanda^22^ were used to predict mRNA-targeted miRNAs. The interactions between circRNA-miRNA and miRNA-Mrna were projected into Cytoscape V2.8^23^ to construct the circRNA-miRNA-mRNA networks.

### Metascape

circRNAs are often co-expressed with their cognate linear (m)RNAs from their host gene loci, and the primary sequences of the mature linear RNAs and circRNAs often fully overlap, except for the unique back splicing junction present in circRNAs. Thus, DEcircRNA enrichment was performed on the host linear transcripts, which was analyzed using the annotation information of circRNA source transcripts based on Gene Ontology (GO) and Kyoto Encyclopedia of Genes and Genomes (KEGG) information on Metascape.^24^ Only terms with an adjusted *p*-value < 0.05 were considered significant. The most significant term (minimum count >3, enrichment factor >1.5, and *p* < 0.01) within a cluster was utilized to represent the top enriched cluster, and enrichment patterns were plotted using a clustered heatmap. Protein–protein interaction enrichment analysis was carried out by integrating the STRING, OmniPath, and BioGrid databases. The densely connected network components were screened using a Molecular Complex Detection (MCODE) algorithm (degree cutoff = 2, k-core = 2, node score cutoff = 0.2, and max depth = 100).

## Results

### High-altitude hypobaric hypoxia-induced hearing impairment and hearing acclimatization

The high-altitude hypobaric hypoxia-induced auditory function was evaluated using the ABR threshold. Compared to Ctrl rats, the ABR threshold was upregulated on Day 30 and downregulated on Day 60 (Supplementary Fig. S1), indicating that the hearing acclimatization process had occurred. Persistent acclimatization was also observed on Days 120 and 180 (data not shown).

### Hearing impairment- and hearing acclimatization-related circRNAs

**Fig. 2:**
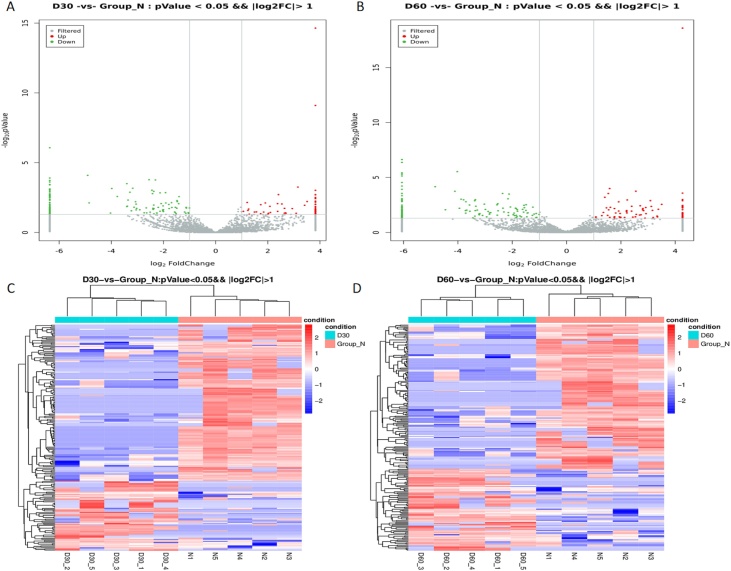
Volcano plot and heatmap of differentially expressed circRNAs (DEcircRNAs). DEcircRNAs at D30 hearing impairment related, (A) and D60 (hearing acclimatization related, (B) are represented as volcano plots. Red points (log2FC>1) indicate upregulated circRNAs; blue points (log2FC<-1) indicate downregulated circRNAs. DEcircRNAs at D30 (C) and D60 (D) were hierarchically clustered using Euclidean distance and are represented with heatmaps.

A total of 211 DEcircRNAs (64 upregulated and 147 downregulated; fold change > 2.0, *p* < 0.05) were identified on Day 30 compared with Ctrl (volcano plot; Fig. 2A); these DEcircRNAs were considered hearing impairment-related circRNAs. In contrast, a total of 221 DEcircRNAs (79 upregulated and 142 downregulated; fold change > 2.0, *p* < 0.05) were identified on Day 60 compared with Ctrl (Fig. 2B); these DEcircRNAs were considered hearing acclimatization-related circRNAs (Supplementary File 1). The relevant expression levels of hearing impairment-related circRNAs (Fig. 2C) and hearing acclimatization-related circRNAs (Fig. 2D) were hierarchically clustered with the Euclidean distance and represented with heat maps. Among hearing impairment-related circRNAs, circSNRK, circPTK2, circPRELID2, circZFR, circIPO11, circMORC3, circKCNH1, circWDR27, circDYM, and circCDR1 were categorized as known disease-related circRNAs (Supplementary File 1); among hearing acclimatization-related circRNAs, circIPO11 was shown to be a liver cancer-related circRNA.

### Biological function of hearing impairment and acclimatization-related circRNAs

**Fig. 3:**
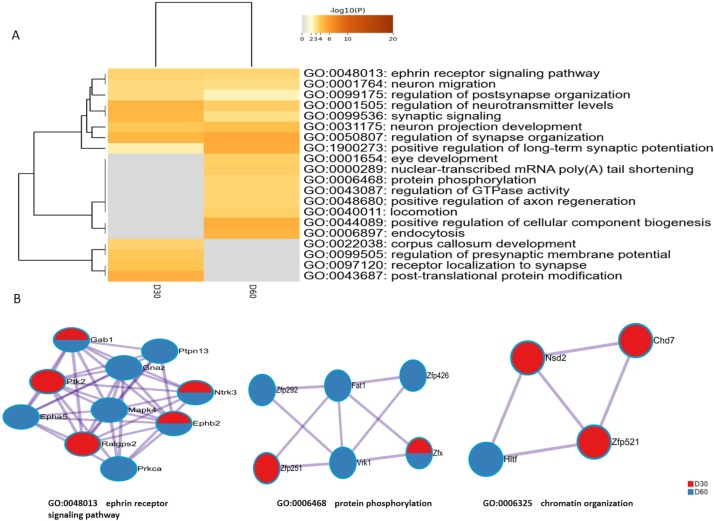
Metascape analysis of stage-related circRNA host genes. (A) Hearing impairment- and hearing acclimatization-related circRNAs were annotated using Metascape with GO and KEGG databases. The top enriched clusters are represented in a heatmap. (B) The Molecular Complex Detection (MCODE) algorithm was used to screen the condensed modules in the stage-related circRNA host genes interaction network.

The host linear transcripts of DEcircRNAs were subjected to enrichment analysis to indicate the potential function of hearing impairment- and hearing acclimatization-related circRNAs using Metascape (Fig. 3A, colored by p-values). It is worth noting that regulation of the presynaptic membrane potential (GO:0099505), receptor localization to synapse (GO:0097120), and post-translational protein modification (GO:0043687) were hearing impairment stage-related processes; positive regulation of axon regeneration (GO:0044089) and endocytosis (GO:0006897) were hearing acclimatization stage-related processes. In contrast, MCODE components identified densely connected complexes of ephrin receptor signaling pathway (GO:0048013), protein phosphorylation (GO:0006468), and chromatin organization (GO:0006325) in both the hearing impairment stage and hearing acclimatization stage (Fig. 3B).

### ceRNA network in high-altitude hypobaric hypoxia-induced hearing impairment

**Fig. 4:**
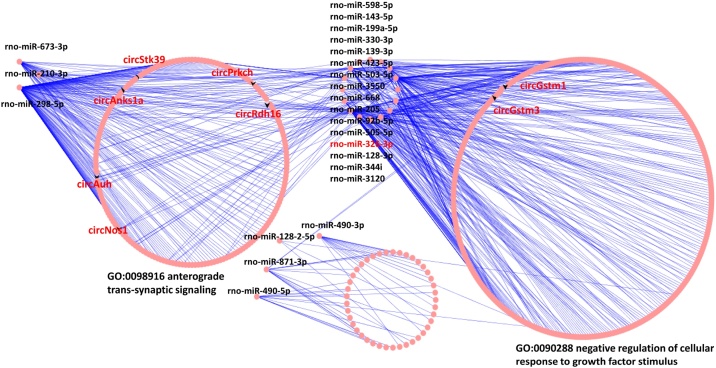
CircRNA-miRNA-mRNA ceRNA network in high-altitude hypobaric hypoxia-induced hearing impairment. The circRNA-miRNA-mRNA ceRNA network was grouped using attributes with eccentricity. The black V represents circRNAs, and the grouped miRNAs are marked in black.

The constructed hearing impairment-related ceRNA network is represented in an eccentricity circle layout (Fig. 4). The eccentricity of a node in a network is the maximum distance to any other node, which could be utilized to indicate the importance of a node within the network. In our analysis, we found that clustered rno-miR-673-3p, rno-miR-210-3p, and rno-miR-298-5p could regulate CircStk39, CircPrkch, CircRdh16, CircNos1, CircAub, and circAnks1a, which are involved in anterograde trans-synaptic signaling (GO:0098916). In contrast, clustered rno-miR-598-5p, rno-miR-143-5p, rno-miR-199a-5p, rno-miR-330-3p, rno-miR-139-3p, rno-miR-423-5p, rno-miR-503-5p, rno-miR-3550, rno-miR-668, rno-miR-205, rno-miR-92b-5p, rno-miR-505-5p, rno-miR-326-3p, rno-miR-128-3p, rno-miR-344i, and rno-miR-3120 could regulate circGstm1 and circGstm3, and the primary function was negative regulation of cellular response to growth factor stimulus (GO:0090288). Collectively, these results demonstrated that anterograde trans-synaptic signaling and negative regulation of cellular response to growth factor stimulus are involved in the hearing impairment stage.

### ceRNA network in high-altitude hypobaric hypoxia-induced hearing acclimatization

**Fig. 5:**
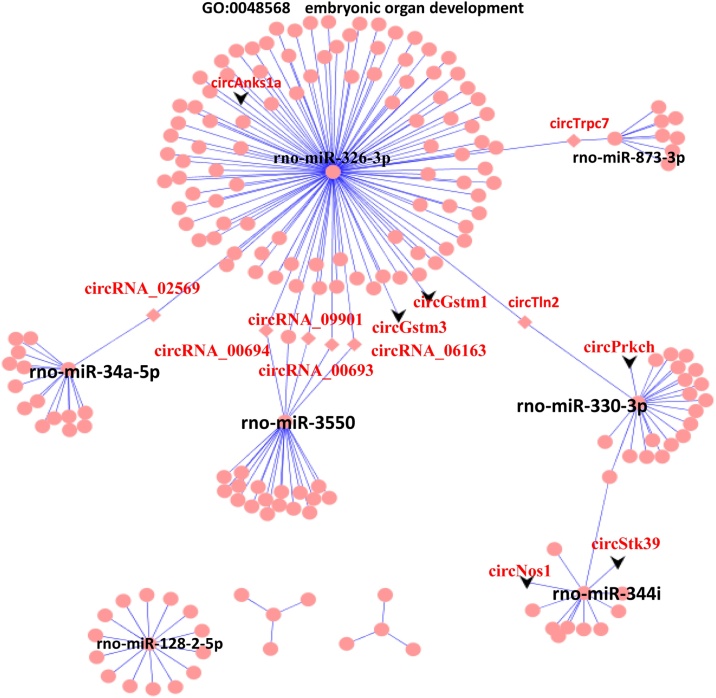
CircRNA-miRNA-mRNA ceRNA network in high-altitude hypobaric hypoxia-induced hearing acclimatization. The circRNA-miRNA-mRNA ceRNA network was grouped using attributes with eccentricity. The black V represents circRNAs indicated in Fig. 4, and circRNAs (degree > 2) are labeled as diamonds.

The hearing acclimatization-related ceRNA network is also represented in an eccentricity circle layout (Fig. 5). Rno-miR-326-3p could regulate cirTrpc7, circTln2, circRNA_00693, circRNA_09901, circRNA_00694, and circRNA_02569, which are involved in embryonic organ development (GO:0048568). In contrast, rno-miR-326-3p, rno-miR-330-3p, rno-miR-344i, rno-miR-128-2-5p, rno-miR-3550, rno-miR-34a-5p, and rno-miR-873-3p were hub microRNAs in the ceRNA network. It is worth noting that in the ceRNA network of hearing impairment (Fig. 4), CircStk39, CircPrkch, CircNos1, circAnks1a, circGstm1, and circGstm3 were identified as the hub node (shape as V), whereas in the ceRNA network of hearing impairment (Fig. 5), circRNAs were located as edge nodes (shape as V).

## Discussion

In this study, rats were airlifted from plain to high altitude positions to induce high-altitude hypobaric hypoxia-associated hearing impairment and hearing acclimatization. Hearing impairment- and hearing acclimatization-related circRNAs and ceRNAs were identified. Functionally, our analyses revealed that anterograde trans-synaptic signaling (GO:0098916) and negative regulation of cellular response to a growth factor stimulus (GO:0090288) might contribute to hearing impairment, and embryonic organ development (GO:0048568) could lead to hearing acclimatization. It is worth noting that anterograde trans-synaptic degeneration occurs after optic neuritis and promotes the progression of hearing impairment.^20^ Brain-Derived Nerve growth Factor (BDNF) plays a vital role in the development of the cochlear, and intracochlear BDNF administration has been suggested to improve hearing in guinea pigs.^25^ In the middle ear cholesteatoma, keratinocyte growth factor can activate p63 to modulate epidermal progenitor cell kinetics.^26^ These findings indicate that such processes might also be involved in high-altitude hypobaric hypoxia-induced hearing impairment and acclimatization. Together, these findings provide a theoretical basis for circRNAs as biomarkers and therapeutic targets in high-altitude hypobaric hypoxia-associated hearing impairment and hearing acclimatization.

Network structures are ubiquitous in genomics biology, and network eccentricity can be utilized to evaluate the magnitude properties of each node.^27^ In this study, we found that the same circRNAs (circStk39, circPrkch, circNos1, circAnks1a, circGstm1, and circGstm3) may demonstrate hub eccentricity in the hearing impairment process, while demonstrating edge eccentricity in the hearing acclimatization process. This indicates that eccentricity structure-related epigenetic regulation might have an essential role in hearing impairment and hearing acclimatization processes. Mechanically, circAnks1a can enhance the interaction between transportin‐1 and Y‐Box‐Binding protein-1 (YBX1) and facilitate YBX1 nuclear translocation. Subsequently, in the nucleus, circAnks1a can combine with the Vascular endothelial growth factor B (Vegfb) promoter and recruit YBX1 to the Vegfb promoter to enhance Vegfb transcription.^28^, ^29^ On the other hand, cytoplasmic circAnks1a can sponge miR‐324‐3p to regulate Vegfb expression to mediate functional vasculature restoration in hearing loss.^30^ These findings indicate a potential circAnks1a/miR‐324‐3p/Vegfb pathway in the hearing impairment process, which may mediate the transition into the hearing acclimatization process.

In the hearing acclimatization process, circTln2 and cirTrpc7 were found to be the hub circRNAs, and miR-873-3p, miR-330-3p, miR-344i, miR-128-2-5p, miR-34a-5p, and miR-3550 were the hub microRNAs. Mechanically, Tln2 can encode talin 1-related protein, which assembles actin filaments to promote the spread and migration of fibroblasts and osteoclasts.^31^ Transient receptor potential isoform 7 (Trpc7) enriches in striatal regions, depolarizes the membrane potential, activates or inactivates voltage-gated Ca2+ channels, and regulates multiple cellular functions.^32^, ^33^, ^34^ miR-873-5p and miR-330-3p can regulate PIK3/AKT/mTOR, NF-κβ, Wnt/β-catenin, and MEK/ERK signaling pathways to affect cancer cell proliferation, migration, invasion, stemness, and glycolysis.^35^, ^36^ miR-34a-5p can suppress PTEN-induced kinase-1 (Pink1) expression to inhibit the Pink1-mediated mitophagy process.^37^ Together, these results demonstrate that cytoskeletal biomechanics and membrane electrophysiology may be exciting areas of future research focused on hearing acclimatization processes.

As a known disease-related circRNA, circIPO11 has been reported to be involved in both hearing impairment and hearing acclimatization processes. CircIPO11 can interact with miR-106a-3p, miR-424-5p, and miR-659-3p or recruit DNA Topoisomerase I (TOP1) onto the GlI Family Zinc Finger 1 (Gli1) promoter to activate hedgehog signaling to drive self-renewal of liver cancer-initiating cells.^38^, ^39^, ^40^ However, the role of circIPO11 in cochlea tissue-related hearing impairment and hearing acclimatization requires further analysis.

On the other hand, miR-326-3p and miR-128 were identified as the hub nodes in both hearing impairment- and hearing acclimatization-related ceRNA networks. MiR-326-3p can target FcγRIII to ameliorate high glucose and oxidized low-density lipoprotein immune complex-induced fibrotic injury in renal mesangial cells.^41^ In breast cancer, miR-326 inhibits the ErbB/PI3K pathway to suppress proliferation and invasion, which can serve as a potential therapeutic target.^42^ MiR-326-3p mimics have been shown to accelerate apoptosis and senescence of skin fibroblasts.^43^ miR-128 can regulate both bone marrow-derived mesenchymal stem cells and osteoblast-related bone metabolism. On the other hand, miR-128 also participates in the regeneration of skeletal muscles by targeting myoblast-associated proteins.^44^ Thus, studying the roles of miR-326-3p and miR-128 in hearing impairment and hearing acclimatization is an exciting field. CircRNA-miRNA-mRNA regulatory network interpretation will be a useful tool to reveal the specific bioprocesses and molecular targets that can be attributed to high-altitude hypobaric hypoxia-induced hearing impairment and hearing acclimatization.

Some limitations of our study should be acknowledged. CircRNAs exert biological functions by acting as transcriptional regulators, microRNA sponges, and protein templates.^45^, ^46^, ^47^, ^48^ However, this study only focuses on the potential sponge function of circRNAs. In addition to noncoding RNAs, natural circRNAs were recently discovered to be protein-coding RNAs, and thus the effects of protein-coding circRNAs identified in this study point to an exciting new field of research. Due to the limited availability of suitable datasets about circRNA, miRNA, and mRNA expression in high-altitude hypobaric hypoxia-induced hearing impairment and acclimatization, we encountered challenges in confirming the results. Furthermore, the results in this study were obtained using bioinformatics analyses, and some RNA binding experiments, RNA sequencing, and cell function experiments should be used to further explore the relationship between ceRNA networks and high-altitude hypobaric hypoxia-induced hearing impairment and acclimatization.

## Conclusion

This study is the first to investigate and decipher key circRNAs and circRNA-miRNA-mRNA ceRNA network contributing to high-altitude hearing impairment and hearing acclimatization. Our framework methodology provides a valuable tool for identifying circRNA-mediated regulation in different stages of hearing impairment and acclimatization.

## CRediT authorship contribution statement

Formal analysis: MTH, THW, QZDJ and XZL; Project administration: LZGG; Data analysis: HLR, KZDZ, BHZ, and XZL; Supervision: LZGG; Writing original draft: DZAW. All authors read and approved the final manuscript.

## Consent for publication

Not applicable.

## Statement of ethics

The entire experimental protocol was approved by the Medical Ethics Committee of Tibet Fukang Hospital (No. 2018-XZDX-006) and conducted in accordance with the ARRIVE guideline. All methods were performed in accordance with the relevant guidelines and regulations.

## Funding

This work was supported by grants from the 10.13039/501100001809National Natural Science Foundation of China (No. U22A20340; No. 82060588; No. 81860567), The key research and development plan of the Tibet Autonomous Region Science and Technology Program (No. XZ202301ZY0011G), Natural Science Foundation of Tibet Autonomous Region (No. XZ202001ZR0040G). The academic development support project for young doctors of Tibet University (No. zdbs202216).

## Data availability statement

All data generated or analyzed during this study are included in this published article (and its Supplementary information files).

## Declaration of competing interest

The authors declare no conflicts of interest.
