# Supplementary material for: CircRNA-miRNA-mRNA regulatory network in high-altitude hypobaric hypoxia-induced hearing impairment and hearing acclimatization
Source: Braz J Otorhinolaryngol. 2025 Jan 27;91(2):101557. doi: 10.1016/j.bjorl.2024.101557 (PMC11808620; doi:10.1016/j.bjorl.2024.101557)
Supplement: Supplementary file 1 [file mmc1.docx]

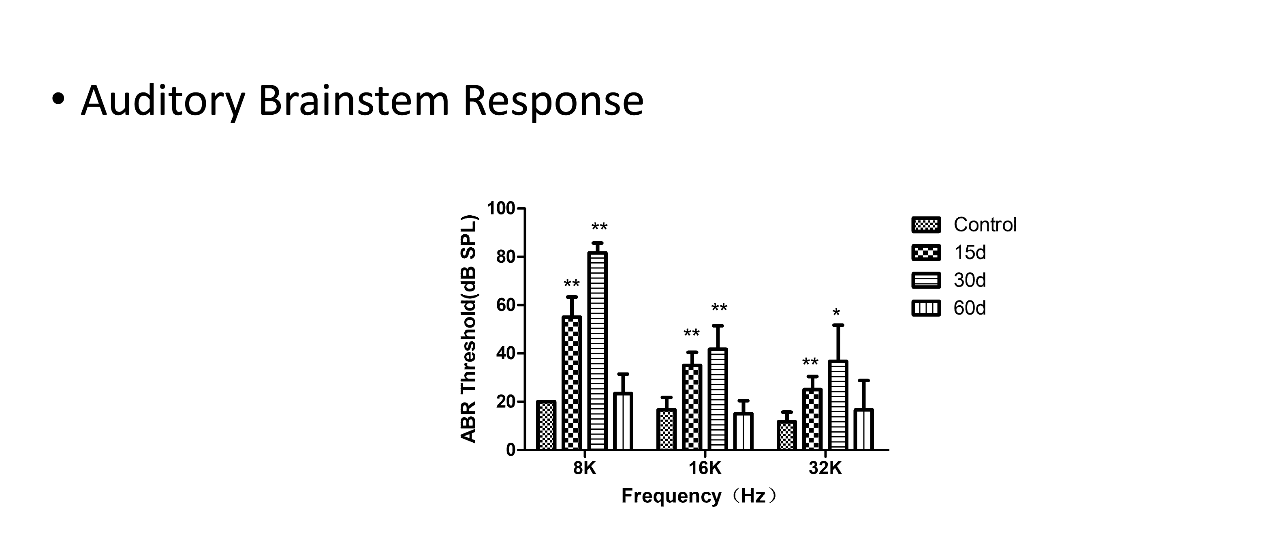


**Supplementary Figure 1. ABR measurements of airlifted Wistar rats from plain to plateau with indicated time at 8, 16, and 32 kHz.** Hearing acclimatization was determined using the auditory brainstem response (ABR) test. Day 0 was utilized as control. The data were presented as mean ± SD. ***p* < 0.01 compared to control, **p* < 0.05 compared to control.
